# Policy maker and health care provider perspectives on reproductive decision-making amongst HIV-infected individuals in South Africa

**DOI:** 10.1186/1471-2458-7-282

**Published:** 2007-10-05

**Authors:** Jane Harries, Diane Cooper, Landon Myer, Hillary Bracken, Virginia Zweigenthal, Phyllis Orner

**Affiliations:** 1 Women's Health Research Unit, School of Public Health and Family Medicine, Faculty of Health Sciences, University of Cape Town, Anzio Road, Observatory, 7925, Cape Town, South Africa; 2 Infectious Diseases Epidemiology Unit, School of Public Health and Family Medicine, University of Cape Town, Cape Town, South Africa; 3 Department of Epidemiology, Mailman School of Public Health, Columbia University, New York, USA; 4 Population Council, New York, USA

## Abstract

**Background:**

Worldwide there is growing attention paid to the reproductive decisions faced by HIV-infected individuals. Studies in both developed and developing countries have suggested that many HIV-infected women continue to desire children despite knowledge of their HIV status. Despite the increasing attention to the health care needs of HIV-infected individuals in low resource settings, little attention has been given to reproductive choice and intentions. Health care providers play a crucial role in determining access to reproductive health services and their influence is likely to be heightened in delivering services to HIV-infected women. We examined the attitudes of health care policy makers and providers towards reproductive decision-making among HIV-infected individuals.

**Methods:**

In-depth interviews were conducted with 14 health care providers at two public sector health care facilities located in Cape Town, South Africa. In addition, 12 in-depth interviews with public sector policy makers and managers, and managers within HIV/AIDS and reproductive health NGOs were conducted. Data were analyzed using a grounded theory approach.

**Results:**

Providers and policy makers approached the issues related to being HIV-infected and child bearing differently. Biomedical considerations were paramount in providers' approaches to HIV infection and reproductive decision-making, whereas, policy makers approached the issues more broadly recognizing the structural constraints that inform the provision of reproductive health care services and the possibility of "choice" for HIV-infected individuals.

**Conclusion:**

The findings highlight the diversity of perspectives among policy makers and providers regarding the reproductive decisions taken by HIV-infected people. There is a clear need for more explicit policies recognizing the reproductive rights and choices of HIV-infected individuals.

## Background

Worldwide there is growing recognition of the reproductive decisions faced by HIV-infected individuals. Studies in both developed and developing countries have suggested that many HIV-infected women continue to desire children despite knowledge of their HIV status [1-4]. Despite the increasing attention to the health care needs of HIV-infected individuals in low resource settings, little attention has been given to their broader needs such as reproductive choice and intentions [5].

The HIV/AIDS epidemic has far reaching consequences for women's reproductive health care services. South Africa is currently experiencing one of the worst HIV/AIDS epidemics in the world with a national HIV prevalence rate of 30% [6]. Here and in many other countries of sub-Saharan Africa, there are a sizeable proportion of young women at the beginning of their reproductive lives, who are infected with HIV, presenting a major challenge to the delivery of reproductive health care services.

Providers play a crucial role in determining access to, and quality of, reproductive health services [7] and their influence is likely to be heightened in delivering services to HIV-infected women [5,8]. The perception that HIV-infected women should not engage in sexual relationships or have children could compromise health care services and impinge on HIV-infected individuals' reproductive rights and choice [2,8]. Furthermore, providers may promote specific services such as sterilization or abortion and compromise or limit women's reproductive choices.

Research in South Africa and elsewhere has demonstrated the important roles that health service providers play in determining women's access to reproductive health services generally, and it is likely that their influence may be particularly important in the case of HIV-infected women [7,9-11]. Reproductive health policy changes in South Africa after 1994 gave recognition to human rights including sexual and reproductive rights [12]. Policy makers within the sphere of reproductive health will continue to play a pivotal role in new policy and guidelines as they relate to the health care needs of HIV-infected individuals.

This article reports on qualitative research that explored health care providers, public sector health policy makers and representatives of non-governmental organizations (NGOs) attitudes towards HIV-infected individuals' fertility intentions and reproductive choices. The research formed part of a broader study in which HIV-infected women and men were interviewed about their reproductive intentions and is reported elsewhere [13]. Understanding providers and policy makers' attitudes is key as they are likely to play important roles in the shaping and delivery of reproductive health care services. It is intended that this research will inform the development of policy and programmes to support reproductive decision-making and choice among HIV-infected individuals in South Africa.

## Methods

### Study site

The study was conducted between May 2004 and January 2005 at two public sector primary health care clinics in Cape Town, South Africa. Both health care facilities are located in predominantly black urban working class communities, with a high HIV prevalence and are broadly representative of the types of services available for HIV-infected individuals in this setting.

### Study design

In-depth interviews were conducted with 14 health care providers delivering HIV care or anti-retroviral (ARV) treatment at two study sites. Health care providers consisted of 3 public health doctors, 7 registered professional nurses and 4 HIV adherence counselors. An additional 12 in-depth interviews were conducted with public sector policy makers and managers, and managers within HIV/AIDS and reproductive health NGOs. Policy makers included high-level managers and directors working in reproductive and sexual health, and HIV/AIDS directorates in Provincial level (Western Cape) Departments of Health. Key influential people from the NGO sector included local and international advocacy and service delivery organizations involved in HIV/AIDS and reproductive and sexual health. In South Africa, the NGO sector has over the past ten years assisted the government in the treatment, support and care of HIV-infected individuals.

Respondents were recruited using purposive sampling. All providers who worked at the two health care facilities, from which we recruited HIV-infected women for the larger study, were contacted and invited to participate in the study. All in-depth interviews were conducted in English by fieldworkers trained in qualitative research methods. All interviews were audio tape-recorded and transcribed verbatim. Informed consent was obtained from all participants prior to recording. Interviews lasted approximately 45 minutes and were conducted in a private space within the respondents' place of work. Interview guides were semi-structured, open-ended and probing. Areas explored in the interview guide included: policy makers and providers attitudes towards HIV-infected women's reproductive intentions and the role of male partners in the process; health service delivery factors influencing reproductive choice including the impact of availability of ART(anti-retroviral treatment) on reproductive choice and the management of pregnancy and contraceptive provision for HIV-infected women.

### Data analysis

Data were analysed using a grounded theory approach, based on a process that helps researchers to "discover" categories, themes and patterns that emerge from the data [14]. Initial categories for analysing data were drawn from the interview guides [15], and themes and patterns emerged after reviewing the data. The computer software package ATLAS ti. was used to facilitate sorting and data management (Scientific Software Developments, 1997). Over several meetings, members of the research team developed and refined the codes using the key issues probed. The transcripts were coded by the research team and then cross checked for coder variation. The data was then reviewed for major trends and crosscutting themes were identified and issues for further exploration were prioritized for final analysis. Coding discrepancies were resolved through discussion and consensus from all research team members.

All participants provided written informed consent. Confidentiality and anonymity was ensured by removing all identifiers including participants' names and places of work from the data. Permission was obtained from the local health authority to interview providers at their facilities. The University of Cape Town's Research Ethics Committee and the Population Council's Institutional Review Board granted approval to conduct the research. Individual data has been kept confidential.

## Results

Fourteen health care providers and twelve policy makers were interviewed. The mean age of providers was 38 years (range 22 – 69 years), 79% (n = 11) were women and 21% (n = 3) were men. The mean age of policy makers was 47.8 years (range 40 – 60 years), 67% (n = 8) were women and 33% (n = 4) were men.

Health care providers and policy makers approached the issues related to being HIV-infected and child bearing differently. Biomedical considerations were paramount in providers approach to HIV infection and reproductive decision-making, whereas, policy makers tended to approach the issues more broadly recognizing the structural constraints that inform the provision of reproductive health care services and the possibility of "choice" particularly with regards to HIV-infected individuals.

### Attitudes towards HIV-infected individuals reproductive intentions

#### Biomedical concerns

Health care providers' attitudes towards HIV-infected women's fertility intentions were largely shaped by their experiences in individual patient care. Providers tended to see the issues in terms of medical concerns, particularly in terms of the potential impact of a pregnancy on HIV disease progression. Many providers felt that deciding to have a child required careful planning and consideration, with a "right time" to fall pregnant, which included an adequately high CD4 count, access to ART and PMTCT programmes, and whether the individual was physically healthy. Explained by a doctor:

*We never tell people they can't have children ... a lot of people say "if you have HIV what are you doing having a baby?" But you have to recognize that people are entitled to have children and that is their choice, but we would prefer them to have their own health sorted out first*. (Doctor)

However, providers also considered a woman's socio-economic circumstances and strong desire to experience motherhood when making recommendations. A nurse explored the complex circumstances faced by some women:

*Looking at the patient, one considers, can you afford to have this baby? Are you well? Are you on ARV's? ...What is this baby going to do to your life? Is it going to make it better, is it going to make it worse? ... That's the way I look at it forgetting about their positive status. They want to prove they can have children*. (Nurse)

#### Reproductive Choice

Providers acknowledged that their counselling of patients about their reproductive options depended, in part, on the patient's health status. Despite this, almost all providers were familiar with the principles of reproductive rights and acknowledged the importance of "a woman's right to choose". However, some providers acknowledged that choice was often a more complex issue, alluding to the importance of remaining "objective and non-judgmental", and allowing women to make "informed choices" even though [it] might not be the "right choice".

Similarly, policy makers focused on issues of reproductive choice and felt that HIV-infected women's fertility intentions could be a politically sensitive issue. Concern was raised that suggesting women curtail or delay having children could be interpreted as infringing on women's reproductive rights and choice as enshrined in the South African constitution. Notwithstanding this, many acknowledged the difficulties of offering reproductive choice in an environment of limited health care resources and a burgeoning HIV/AIDS epidemic. As a policy maker explained:

*South Africa is different to first world countries where they are able to offer women various services during a pregnancy such as delivery with an elective Caesar ... and sperm washing which is kind of irrelevant in our setting*. (NGO policy maker)

Some public health care sector policy makers also expressed concerns about the feasibility of reproductive choice in a context in which there were an increasing number of AIDS orphans and overstretched treatment services for HIV-infected people.

#### Importance placed on motherhood

Some providers and public sector policy makers acknowledged societal expectations regarding childbearing and that some women faced pressure from boyfriends or husbands to have children, fearing abandonment if they did not.

Nurse providers cited numerous reasons why HIV-infected women may want to have a baby. These included: wanting to have "something of their own", pressures from partners, in-laws and family to have children, a woman's age and whether they already had children. A nurse provider suggested that younger women or those who had no children often wanted at least one child, whereas women who already had children might not have the same desires. Furthermore, women who were economically dependent on their male partners often felt pressured into having a baby so as to remain in the relationship.

Providers grappled with the complexities involved in providing unbiased advice to HIV-infected women around childbearing. Nevertheless, some providers displayed insight into the emotional difficulties faced by women who were diagnosed HIV positive and what this in turn could mean for future fertility intentions. A nurse described the dilemmas faced by a young HIV-infected woman of seventeen who she had counselled. Whilst on the one hand she could empathise with the client's reproductive intentions, on another level, she felt it was inappropriate to have a child due to the perceived difficulties associated with both an adolescent pregnancy and being HIV-infected.

*" I am going mad ...I must have a baby" I could empathize with her... for some reason in my experience this urge just comes up and there is very little you can do about it...So I said to her, " look, exclude the fact that you are HIV [positive], at 17 do you need a baby right now?" I still told her the choices, but she was saying to me, "I know it's not right but I can't help this"*. (Nurse)

Another nurse provider acknowledged that there was little she could do to prevent HIV-infected women from wanting children despite the perceived health risks, asserting she was "at peace with decisions that people make and that one can't make decisions for people, nor should one try to change things beyond one's control". Similarly, providers and policy makers spoke of the importance of motherhood in many of the communities in which women lived. Having a child in this context was frequently a defining feature of womanhood.

### Role of men

Both providers and policy makers alluded to the difficulties associated with including men in reproductive health care services including fertility-related counselling and contraception. Some providers stated that men's involvement in contraceptive decision making processes was minimal, contraception and more general reproductive matters were viewed by men as part of a "woman's responsibility".

A public health policy maker felt that reproductive health services were not "gender sensitive", as they focused more on women's needs and in the process left men "on the outside". In relation to contraceptive services and dual protection he had this to say:

*I think the contraceptive services have always been geared more towards women than men of course,... men are not taking their equal share of responsibility for fertility regulation. And I think maybe the staff are also not so good at handling men and their feelings about contraception and so on*. (Policy maker)

Indeed, several providers were open about their limited knowledge of HIV-infected men's reproductive intentions and their relative inexperience with male clients given the relatively small number of men who sought treatment for HIV/AIDS at their health care clinics.

### Health service delivery factors influencing reproductive choice

Discussion of reproductive health services for HIV-infected individuals led many policy makers and providers to speak more generally about the structural constraints endemic to the healthcare system as a whole. Several providers identified systemic problems that inhibited the public healthcare system from providing services to all patients, regardless of HIV status. These included the general organization of existing health services, including a lack of integration between HIV and reproductive healthcare services. A policy maker referred to the current system as "vertical protocol-driven silos" that inhibited referral systems between different services. Furthermore, overall staff shortages and associated low morale inevitably influenced the quality of HIV-related services provided. A policy maker explained:

*There is the philosophy and then there's reality...sometimes I sit in ante-natal clinics and I hear some of the exchanges that go on between staff and patients and they fall very short of the ideal. They [healthcare staff] are tired, discouraged, depressed, and overwhelmed*. (Public Health policy maker)

Related to this, providers identified difficulties with the delivery of health care services, including ART, the management of HIV and pregnancy and appropriate contraceptive provision and counselling.

#### Impact of ART on reproductive intentions

Several providers acknowledged that the availability of ARV treatment made possible an extended lifespan and may cause some HIV-infected women and men to rethink their reproductive options. A doctor explained that after several months on medication patients began to feel well and that they were "not going to die, but live, and part of living is about reproducing and having kids".

Providers recognized the importance of encouraging greater openness in discussing reproductive choices, particularly amongst pregnant women. Some doctors feared that if providers did not acknowledge patients' pregnancy intentions and counsel them accordingly, they may cease seeking ARV treatment. A doctor explained:

*Of course there definitely is a time and a place for avoiding getting pregnant with ARV's...But it shouldn't come across in patients' minds that being on ARV's, that you can never have children. I think that will stop people from coming for treatment*.(Doctor)

Providers also described the possible difficulties in managing a pregnancy in a woman taking ARV treatment. This was further underscored by a lack of specific clear guidelines available to providers on reproductive health counselling for both pregnant women and those wanting to become pregnant. Several providers emphasised the need for women to be open about their fertility intentions in order to limit the risk to both the woman and her infant. Given the possible teratogenic effects of the antiretroviral therapy Efavirenz, providers were particularly concerned about the risk of an unplanned pregnancy for women currently undergoing ART.

#### Contraceptive provision and counselling

Both policy makers and providers identified a lack of clear policy guidelines and training around contraceptive counselling for HIV-infected individuals as a shortcoming in current reproductive health care services. Providers felt that they had insufficient knowledge of the possible interactions between different ARV treatment regimens and hormonal contraceptives. In the absence of specific contraceptive guidelines, providers developed their "own" guidelines which were often not based on clinical evidence. Contraceptive options suggested for HIV-infected women included injectable contraceptives and to a lesser extent, combined oral contraceptive pills, intra-uterine devices and male condoms. Sterilization was considered suitable for women who already had a child or in the post partum period. Yet some policy makers alerted to the possible dangers of "forced sterilization" of HIV-infected women. Policy makers also voiced concern around continued "vertical family planning services" with a high reliance on injectable contraceptives as this had implications for reproductive counselling and contraceptive choice. A lack of integration between reproductive health care services and HIV care and treatment services was of concern to both policy makers and providers.

Policy makers recognized providers' concerns about a general lack of reproductive health guidelines for HIV-infected individuals. Most policy makers felt that developing counselling guidelines on reproductive options for HIV-infected individuals would be valuable. Without these guidelines providers would continue "bumbling along" and make decisions in an "ad hoc" manner. However, a few expressed reservations about designing reproductive health guidelines specifically targeting HIV-infected individuals, as a large number of peoples' HIV status was unknown and favoured focusing on improving reproductive health services for all.

## Discussion

This study presents valuable new insights into providers' and policy makers' views towards HIV-infected individuals' fertility intentions in South Africa and is the first known study in South Africa to explore these issues. Policy makers and providers reflected on a number of issues that would influence their approach to HIV-infected individuals' fertility intentions and desires. While most participants were aware of the broader social issues that made reproductive decision-making complex for HIV-infected women, their own attitudes towards childbearing in the context of HIV were strongly mediated by biomedical and health service related factors. These included HIV-infected patients' health status, possible health risks associated with pregnancy and access to ART and contraceptive services and an overall lack of clear reproductive health policy guidelines for HIV-infected individuals.

Whilst discourse around reproductive rights and choice emerged spontaneously in both the providers and policy makers discussions, there was little consideration of how it would translate into every day practice. Moreover, "reproductive choice" was not fully interrogated by providers in terms of what choice really means for women in situations of inequitable gender relations and societal norms which view reproduction as an integral part of women's lives and underscores the difficulty in translating rhetoric into practice. Recognition by many policy makers and providers towards the central role of childbearing on women's social identities was superseded by concerns of an overburdened health care system.

For many providers and policy makers an overriding concern was an overextended health care system with limited capacity to provide adequate reproductive health care services to a general population of women and men using public sector services, let alone to HIV-infected individuals. This is not surprising considering the flux in the health care system in South Africa and controversy surrounding the relatively slow introduction of ARV treatment programmes, with confusing and contradictory messages to both providers and the public regarding the efficacy and safety of ARV treatment [12]. Within this context, support for HIV-infected individuals' reproductive rights and choice were often guarded and ambiguous. While providers stated they respected HIV-infected women's reproductive rights, it was clear that they felt substantial reservations about HIV-infected women having children.

Policy makers and some providers acknowledged that choice was often not possible, particularly in a climate of judgmental and negative attitudes displayed by healthcare providers, making opportunities for non-judgemental counselling around reproductive choice difficult. Quality of care is not an unexpected issue to emerge and has been reported elsewhere in South Africa within the context of abortion and family planning services [10,11,16]. This situation underscores the need to destigmatize the issues around HIV and childbearing. The potential for providers to shape access to reproductive health services points to the need for health systems interventions which focus on providers [5,7]. Values clarification programmes aimed at distinguishing personal views from professional responsibilities could play an important role in improving the quality of care and reproductive health outcomes of HIV-infected women and men.

Both policy makers and providers were mindful of the dearth of policy and guidelines for dealing with reproductive choice among HIV-infected individuals. World Health Organization guidelines on contraception for HIV-infected individuals exist and appropriate guidelines need to be developed and adapted for reproductive counselling of HIV-infected individuals in South Africa.

A possible limitation to this study is that it was specific to one setting in South Africa and although is broadly representative of prevailing attitudes in South Africa, further research is needed in both South Africa and other resource limited settings.

Moreover, the findings reflect the views of a particular group of health care providers, i.e., providers located within a well resourced urban area with better health care infrastructure in terms of HIV treatment and care than health care facilities located within rural areas of South Africa.

## Conclusion

The results of this study underscores the need for creating linkages between HIV care and treatment and reproductive health care services for HIV-infected individuals in South Africa. Ideas for further research and interventions should include explicit policies that recognise reproductive choice in HIV-infected individuals including improved access to contraception and other reproductive health care services. Training of health care providers to deal sensitively with the reproductive health care needs of HIV-infected individuals from both a psychosocial and biomedical perspective should be initiated. The latter should include potential interactions between ART and hormonal contraceptives and the impact of HIV and ART on pregnancy. The need for integration of all aspects of reproductive health care services with other HIV care and treatment in South Africa needs to be explored.

## Competing interests

The author(s) declare that they have no competing interests.

## Authors' contributions

JH assisted in designing the study, data collection and data analysis and drafted the manuscript.

DC conceptualized and assisted in designing the study, data collection and analysis and critically reviewed the manuscript.

LM and HB conceptualized and assisted in designing the study and data analysis and critically reviewed the manuscript.

VZ conceptualized and assisted in designing the study, data collection and analysis and reviewed the manuscript.

PO assisted in data collection and analysis and reviewed the manuscript.

All authors read and approved the final manuscript.

## Pre-publication history

The pre-publication history for this paper can be accessed here:


